# Weight Status and Differences in Mobility Performance, Pain Symptoms, and Physical Activity in Older, Knee Osteoarthritis Patients

**DOI:** 10.1155/2014/375909

**Published:** 2014-05-25

**Authors:** Matthew J. Garver, Brian C. Focht, Justin Dials, Mark Rose, Alexander R. Lucas, Steven T. Devor, Charles F. Emery, Kevin V. Hackshaw, W. Jack Rejeski

**Affiliations:** ^1^Student Recreation and Wellness Center (SRWC), Abilene Christian University, Room 259, ACU Box 28084, Abilene, TX 79699-8084, USA; ^2^The Ohio State University, Health and Exercise Science Room A36, 305 West 17th Avenue, Columbus, OH 43210, USA; ^3^Capital University, Battelle Hall Room 305, 1 College and Main, Columbus, OH 43209, USA; ^4^The Ohio State University, 145 Psychology Building, 1835 Neil Avenue Mall, Columbus, OH 43210, USA; ^5^The Ohio State University, 480 West 9th Avenue, Columbus, OH 43210, USA; ^6^Health and Exercise Science Department, Wake Forest University, P.O. Box 7868, Winston-Salem, NC 27109, USA

## Abstract

Knee osteoarthritis (OA) is a leading cause of functional disability among American adults. Obesity is a strong independent risk factor for OA. While research emphasizes the role of obesity in the OA-physical function relationship, the extent to which weight status impacts salient physical, health, and pain measures in older, knee OA patients is not well delineated. The primary aim of this study was to assess differences in mobility performance (stair climb and 400-meter walk), mobility-related self-efficacy, pain symptoms (WOMAC), and measures of accelerometer-determined physical activity (PA) as a function of weight status. Analysis of covariance was conducted to examine differences on the dependent variables. Obese class III patients were outperformed by their counterparts on nearly every measure of mobility, mobility-related self-efficacy, and the assessment of pain symptoms. These outcomes did not differ among other weight comparisons. Normal weight subjects outperformed classes I, II, and III counterparts on most measures of PA (engagement in moderate or greater PA and total weekly steps). Additionally, overweight participants outperformed obese class II participants and obese class I participants outperformed obese classes II and III participants on total weekly steps. Collectively, these findings underscore the meaningful differences observed in relevant OA outcomes as a function of increasing levels of body weight.

## 1. Introduction

Knee osteoarthritis (OA) is a leading cause of functional disability among American adults [1]. Joint damage and the accompanying pain symptoms of knee OA are postulated to serve as the primary causes of activity restriction and difficulty in the performance of tasks involving ambulation and transfer [2, 3]. Age and obesity are strong independent risk factors for arthritis [4]. Data summarized from the 2005 Behavioral Risk Factor Surveillance System found the national prevalence for arthritis to be 31.59% but individuals in the obesity classes were clearly more at risk for having arthritis compared with those in normal or overweight classifications according to body mass index (BMI) standards [4]. Given that the prevalence of obesity continues to rise, delineating the role of body weight on OA outcomes is important in guiding the design and delivery of self-management interventions.

Emerging findings underscore the potential role of body weight in the OA-physical function relationship. For example, obesity has been associated with locomotor disability in men and women with lower limb OA [5]. Moreover, data from the mechanical factors in arthritis of the knee trial, a 3-year trial employing 285 knee OA patients, indicated a significant difference in the baseline BMI of subjects with the most favorable (average BMI 29.9) and worst (average BMI 31.6) WOMAC outcomes [3]. More recently, Okoro et al. found that class III obesity was associated with self-reported disability outcomes in those with (and also without) arthritis [6]. Odds ratios for disability were also higher for the patients in the overweight and obesity classes compared with normal weight subjects [6]. It is important to note that assessments of height, weight, disability, and presence of arthritis were all obtained via self-report, thereby considerably limiting what can be concluded about the relationships among these outcomes.

Despite these data, Schoffman and colleagues suggest that “no studies to date have examined the association between BMI and objectively measured laboratory-tested physical function measures.” Hence, their data represents an important addition to the literature. Schoffman and colleagues reported that an unhealthy weight escalates unfavorable outcomes on physical function (six-minute walk distance, stair-climb ability, sit and reach flexibility, and walk speed) and health-related (pain symptoms, fatigue, disability, and quality of life) outcomes in a range of arthritis patients from 19 to 87 years of age [7]. However, they did not examine differences in these outcomes according to BMI class (normal, overweight, class I, class II, or class III obesity).

There is clearly a need for evidence supporting and extending knowledge regarding the relationship of weight status and both physical function and health-related quality of life outcomes in OA patients. Furthermore, as the prevalence of OA increases with age, the extent to which weight status impacts salient physical, health, and pain measures in older, knee OA patients, specifically, is a pressing public health concern.

Baseline data from the improving maintenance of physical activity in older, knee osteoarthritis patients trial-pilot (IMPACT-P) [8], a 12-month, randomized control trial involving 80 knee OA patients aged 55–84, were used for this study. The primary aim of this study was to examine differences in mobility performance, mobility-related self-efficacy (SE), pain symptoms, and physical activity (PA) in older, knee OA patients as a function of weight status defined via established BMI classifications.

## 2. Methods

### 2.1. Study Approval and Eligibility

IMPACT-P was approved by the Institutional Review Board of The Ohio State University. Participants were recruited from local medical clinics and arthritis foundation aquatics facilities. The full range of inclusion and exclusion criteria has been reported elsewhere [8]. To recruit a sample of older, community dwelling adults with symptomatic knee OA who had self-reported limitations in function but no serious medical contraindications to participation in center- or home-based PA of moderate intensity, the primary inclusion criteria were (a) age > 55 years; (b) physician documented radiographic evidence of mild to moderate (II or III) tibiofemoral OA according to the Kellgren-Lawrence scale; (c) self-reported knee pain on most days of the month; (d) sedentary lifestyle with less than 20 min of structured exercise participation per week over the preceding 6 months; and (e) self-reported difficulty with common functions such as mobility. Primary exclusion criteria were (a) medical conditions that prohibited safe participation in an exercise program; (b) radiographic evidence of knee joint varus or valgus malalignment that, in the opinion of the physician, would make regular physical activity participation unsafe; (c) the inability to walk without a cane or other assistive devices; (d) OA severity > III on the Kellgren-Lawrence scale; or (e) the inability to complete the 12-month study or otherwise be compliant due to personal demands.

### 2.2. Recruitment Flow

A total of 174 individuals were prescreened for eligibility for the IMPACT-P trial and 80 older adults were ultimately enrolled. Among the 94 individuals not enrolled, the majority were excluded due to lacking time or interest (*n* = 45), medical contraindications (*n* = 15), or being too physically active (*n* = 11) at baseline.

### 2.3. Overview of Procedures

Participants completed a baseline screening visit during which all assessments, described below, were obtained. Participants engaged in mobility performance tasks followed by assessment of general and OA-specific health outcomes collected with valid and reliable questionnaire assessments. At the baseline visit, participants were given verbal and written instructions about wearing the Lifecorder accelerometer for the following 7 consecutive days. Participants were instructed to return the accelerometer to the research staff at the end of the 7-day period.

### 2.4. Sociodemographic Variables and Weight Status Classification

Subjects reported age, sex, race, annual household income, and education level achieved. Subject weight was recorded by study staff and subjects were stratified into weight status classifications based on BMI (mass (kg)/ht^2^ (m)) [9]: underweight and normal weight (<18.5–24.99); overweight (25.00–29.99); obesity class I (30.00–34.99); obesity class II (35.00–39.99); and obesity class III (>40.00).

### 2.5. Mobility Performance

Timed stair climb (SC) and 400-meter walk (400 m) tasks were used as objective assessments of participants' mobility performance. Performance was measured as the total time (in seconds) necessary to complete each task. The SC test involved ascending and descending a set of 8 stairs around a centrally located handrail. A 400 m walk test was completed after the SC task on a 20-meter course (1  lap = 40 m). Individuals walked a total of 10 laps at a brisk, self-directed pace. A chair was placed at the 10-meter point equidistant from each end of the course for safety and rest. Participants were instructed to complete the SC and 400 m tasks as quickly as possible while ensuring safety. Standard encouraging comments were given to subjects during each task. Lower times to complete the tasks represent more favorable mobility performance.

### 2.6. Mobility-Related Self-Efficacy

Assessments of mobility-related SE were collected prior to the commencement of each mobility performance test. SE responses were assessed by asking participants to rate their confidence in successfully complete incrementally more challenging amounts of the SC (ascending and descending the stairs 2, 4, 6, 8, and 10 trips) or 400 m (2, 4, 6, 8, and 10 laps around the 20-meter course) walk task. Confidence was rated specific to each level of the task on a ladder ranging from 0 (no confidence at all) to 10 (complete confidence) and SE scores were calculated by summing the responses, taking the average, and multiplying by 10 to yield a score ranging from 0 to 100. Higher scores reflect greater SE in task completion. This hierarchical procedure has been used and validated previously [2].

### 2.7. Pain Symptoms

Self-reported knee pain was assessed with the Western Ontario and McMaster Universities Osteoarthritis (WOMAC) Index subscale. Participants indicated pain symptoms during the previous 48 hours when engaging in 5 specific activity and movement patterns. The scale total was calculated by summing scores for individual activities. Scores range from 0 to 20 with higher scores indicating worse pain. The WOMAC has demonstrated reliability and validity [10, 11].

### 2.8. Physical Activity

Objectively determined PA was assessed with the Lifecorder accelerometer (Lifecorder EX, Suzuken Kenz Inc. Limited, Japan). The device was used to obtain an assessment of PA participation during the 7-day period following the baseline visit. Participants wore the accelerometer during all waking hours, except when showering, bathing, or swimming. In addition to its pedometer function, the Lifecorder provides assessment of minutes of light, moderate, and vigorous PA participation. Consistent with the metabolic demands for the targeted age group [12], the accelerometer was preset to categorize all PA into minutes of light (<3), moderate (3–6), or vigorous (>6) intensity based on metabolic equivalents.

### 2.9. Data Analysis

Analysis of covariance (ANCOVA) controlling for age, sex, education level, and income was conducted to examine weight group differences on all dependent variables (mobility performance, mobility-related SE, pain symptoms, and measures of PA). Least significant difference procedure was conducted as post hoc analysis to determine the presence of significant mean differences, and effect sizes (Cohen's* d*) were calculated. A probability level of 0.05 was chosen* a priori* to denote significance. Of note, 1 participant, classified as underweight with a BMI of 18, was included in the analysis with the normal weight subjects.

## 3. Results

One subject failed to return the baseline accelerometer and was removed from analysis. Characteristics of the 79 subjects comprising the baseline analysis of weight status are displayed in Table 1. The sample was predominantly female (83.5%), most participants reported being graduates of high school, and all reported having OA in at least one other location in the body beyond the knee(s). Approximately 32% were of minority racial background. Subjects were classified in five groups according to BMI: normal weight (*n* = 8); overweight (*n* = 22); obesity class I (*n* = 19); obesity class II (*n* = 17); or obesity class III (*n* = 13).

### 3.1. Mobility Performance

Results of ANCOVA revealed significant main effects for weight status on both SC and 400 m tasks. Normal (*P* = 0.003; *d* = 1.61), overweight (*P* = 0.001; *d* = 1.32), and obesity class I (*P* = 0.001; *d* = 1.05) participants outperformed obesity class III subjects on the SC performance (*F*(4,70) = 4.599, *P* = 0.002). No other significant differences were noted between the weight status groups for SC performance.

**Figure 1:**
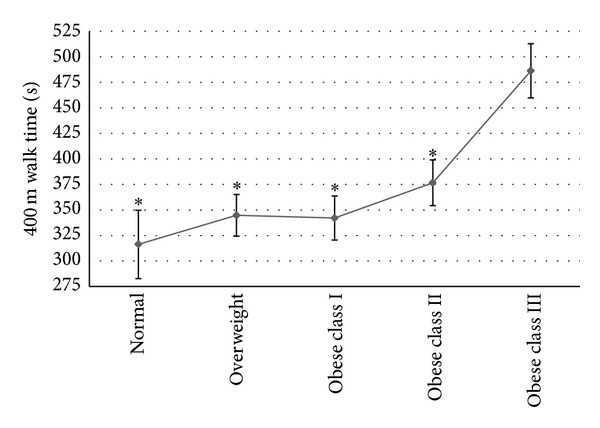
Mean (±SE) 400-meter walk times for participants of different weight status. *Denotes statistical difference from obese class III.

With regard to 400 m walk time, normal (*P* < 0.001; *d* = 1.53), overweight (*P* < 0.001; *d* = 1.25), obesity class I (*P* < 0.001; *d* = 1.03), and obesity class II (*P* = 0.02; *d* = 0.97) participants outperformed obesity class III participants (*F*(4,70) = 6.147, *P* < 0.00). No other significant differences were noted between the weight status groups.  Table 2 displays mean outcomes for mobility performance.  Figure 1 illustrates the 400 m results among the weight status groups.

### 3.2. Mobility-Related Self-Efficacy

No significant effects emerged for stair climbing SE (*F*(4,70) = 1.722, *P* > 0.155). Results of ANCOVA revealed a significant main effect for weight status on walking SE (*F*(4,70) = 4.928, *P* = 0.001). Normal (*P* < 0.001; *d* = 2.28), overweight (*P* = 0.001; *d* = 1.38), obesity class I (*P* = 0.001; *d* = 1.09), and obesity class II (*P* = 0.02; *d* = 1.38) participants had greater walking SE compared with obesity class III subjects. No other significant differences were noted between the weight status groups.  Table 2 displays mean outcomes for mobility performance SE.

### 3.3. Pain Symptoms

Results of ANCOVA revealed significant main effects for weight status on pain symptoms (*F*(4,70) = 2.549, *P* = 0.047). Normal (*P* = 0.007; *d* = 1.52), overweight (*P* = 0.013; *d* = 1.14), obesity class I (*P* = 0.014; *d* = 1.01), and obesity class II (*P* = 0.029; *d* = 1.12) participants had more favorable pain levels compared with obesity class III subjects. There were no other differences to note among the weight status comparisons. The mean outcomes for the WOMAC are displayed in Table 2.

### 3.4. Physical Activity

**Figure 2:**
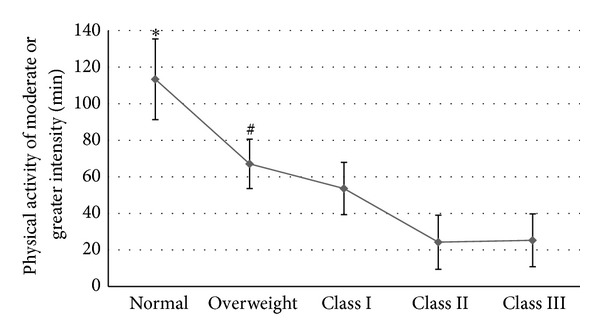
Mean (±SE) engagement in physical activity of moderate or greater intensity for participants of different weight status. *Denotes statistical difference from obese classes I, II, and III. ^#^Denotes statistical difference from obese class II.

Objectively determined PA participation of moderate or greater intensity was assessed using the Lifecorder accelerometer (PA^Mod+^). The analysis of accelerometry also included evaluation of total weekly steps and frequency of bouts of exercise lasting 10 or more minutes in duration. All mean data for the measures of PA are displayed in Table 3. Weight status was found to impact measures of PA determined objectively from accelerometry. Normal weight status subjects engaged in more PA^Mod+^ compared with obesity class I (*P* = 0.025; *d* = 0.80), class II (*P* = 0.001; *d* = 1.14), and class III (*P* = 0.003; *d* = 1.07) counterparts (*F*(4,70) = 3.683, *P* = 0.009) but not those classified as overweight (*P* = 0.083). In addition, overweight counterparts engaged in more PA^Mod+^ compared with obesity class II (*P* = 0.034; *d* = 0.79) counterparts. No other significant differences were noted between the weight status groups for PA^Mod+^.  Figure 2 illustrates the PA^Mod+^ results among the weight status groups.

Weight status was also found to impact total weekly steps. Normal weight status subjects engaged in a greater number of weekly steps compared with overweight (*P* = 0.040; *d* = 1.32), obesity class II (*P* < 0.001; *d* = 1.32), and obesity class III (*P* = 0.001; *d* = 1.32) counterparts (*F*(4,70) = 4.561, *P* = 0.002). The difference in weekly steps between normal and obesity class I subjects is notable (*P* = 0.062) but the comparison failed to reach significance. Overweight subjects engaged in a greater number of weekly steps compared with obesity class II (*P* = 0.030; *d* = 0.81) counterparts. Finally, obesity class I participants engaged in a greater number of weekly steps compared with obesity class II (*P* = 0.021; *d* = 0.56) and obesity class III (*P* = 0.046; *d* = 0.62) subjects. No other significant differences were noted between the weight status groups.

The overall univariate test for frequency of the bouts of exercise lasting 10 or more minutes in duration failed to reach significance (*F*(4,70) = 1.644, *P* = 0.173).

## 4. Discussion

Findings from the present study underscore the meaningful differences observed in relevant OA outcomes as a function of increasing levels of body weight. Notably, consistent with prior findings (Schoffman et al.), baseline data from the IMPACT-P trial demonstrates that mobility performance erodes as weight status increases in knee OA patients [3, 7, 13]. Additionally, the present results extend prior evidence by also demonstrating less favorable mobility-related SE, pain symptoms, and PA participation levels with increasing body weight among older, knee OA patients. Collectively, these findings clearly illustrate the breadth of deleterious effects over which weight status may influence knee OA patients.

Mobility performance and mobility-related SE are integral to preventing disability and loss of independence with advancing age. A strong ability and SE for stair climbing and walking short distances (such as 400 m) have recognizable application for functioning independently. Result from the present study revealed that obese class III patients faired significantly worse than normal, overweight, obese class I, and obese class II patients on mostly all measures of mobility performance and mobility-related SE. Although classes I and II obesity participants outperformed class III patients on mobility performance and related SE assessments, caution is warranted for these subjects with regard to future mobility function. Mean BMI levels of 31.1 and 32.6 have been shown in the literature to correspond to OA persons moving into (BMI of 31.1) and staying in (BMI of 32.6) the lowest levels of physical function over the course of a three-year study [3]. Moreover, there is considerable evidence about the predictive nature of SE in the previous literature with knee OA patients [2, 14]. While SE differences on the SC failed to achieve statistical significance, the collective findings are robust and highlight the multifaceted challenges (functional and psychological barriers) that patients must plan to overcome to take back control of their declining mobility performance.

The time to complete the 400 m walk task provides an overall mean gait velocity. Exploratory analysis revealed mean walking velocities for the normal (1.31 m/s), overweight (1.20 m/s), obese class I (1.12 m/s), obese class II (1.08 m/s), and obese class III (0.81 m/s) participants. Low gait velocities (0.7 m/s) have been found to predict hospitalizations, need for a caregiver, and occurrence of falls in well-functioning persons over 75 [15]. In addition, as a single predictor, gait velocity less than 0.8 m/s has been defined as pathological and associated with impaired ability to rise and walk and impaired ability to stand on one leg [15]. These findings have special relevance for the class III patients in the IMPACT-P study. The data provide support for the role of weight status as a meaningful individual difference that influences risk for loss of mobility in older knee OA patients.

Self-reported pain symptoms of the obese class III participants were significantly worse than all other weight status groups. Indeed, obese class III patients rated their pain symptoms to be 178% higher than normal weight subjects. Moreover, in the first National Health and Nutrition Examination Survey, the presence of knee pain was found to predict difficulty in ambulation, transfer, and performance of instrumental activities of daily living a decade later [16].

Weight status influenced select measures of PA data. Normal weight status subjects engaged in greater amounts of PA (113.4 min) than all other weight status groups except those who were overweight (67.1 min). Additionally, overweight subjects engaged in more PA than obese classes II (24.3 min) and III subjects (25.3 min). The obvious recognition must be made; no group met the recommended level of weekly PA^Mod+^ (150 minutes). Beyond that fact, there was a noticeable gap in PA engagement between normal and overweight subjects and PA engagement for obese class II and class III subjects was woefully inadequate (averaging approximately 3.5 minutes each day). The ability to accumulate weekly steps is very likely impacted by these poor lengths of PA engagement. Indeed, normal weight subjects engaged in greater volumes of daily steps (approximately 7,072) compared with overweight (approximately 4,889), obesity class II (approximately 3,157), and obesity class III (approximately 3,310) subjects. The absolute data about frequency of bouts of exercise lasting 10 minutes or more in duration is concerning despite not being significant statistically. Normal weight subjects, who engaged in double to triple the weekly bouts, only engaged in 3.16 bouts of PA each week that lasted more than 10 minutes in duration.

The cross-sectional nature of this investigation raises questions that cannot be addressed by data from IMPACT-P. For example, do less PA and mobility come before weight gain and OA or does the onset of OA precede mobility challenges and declining PA through a vehicle such as joint pain or loss of strength which leads to obesity? While it might be considered that one occurs before the other, speculatively, both scenarios could operate to the realization of the problems that we see in the literature. Research from Sharma et al. [3] and Cooper et al. [17] supports this speculation. Sharma and colleagues found that low and high levels of activity engagement were linked with poor and more favorable functional outcomes, respectively, in knee OA patients, over a three-year period [3]. In this case, activity engagement (or lack thereof) appears to have led before maintenance (or apparent loss) of function in the OA patients. On the other hand, Cooper et al. found that obesity was predictive of both incidence (*P* < 0.001) knee OA and progression (*P* < 0.05) of knee OA even when considering age and sex in the analysis [17]. In these distinct investigations, the preceding factors and subsequent outcomes were dissimilar.

Irrespective of whether less PA and mobility come before weight gain and OA or whether the onset of OA precedes mobility challenges and declining PA which leads to obesity, the concurrent findings that pain symptoms were exacerbated and PA engagement was reduced in patients of higher weight status exemplify the challenges that clinicians face in combatting this rampant health burden. In addition to declining PA and obesity, factors such as decreased proprioception [18, 19], muscle atrophy [20, 21], ligamentous laxity [17], and previous knee injury [22, 23] might all serve as critical preceding or exacerbating factors which increase the risk for obesity and OA regardless of which develops first.

### 4.1. Limitations and Strengths

As mentioned, the cross-sectional design precludes inference about causal relationships that might exist. The preponderance of females limits application of results to males. In specific cases, the trial may have been inadequately powered to detect differences that may have existed between weight status groups. Also, the trial excluded subjects who were sufficiently active at baseline. Accordingly, the application of the results may not conform to findings on older, knee OA patients who are engaging in more rigorous and lengthy volumes of exercise. Certainly, there are clear connections between obesity and a number of clinical markers with regard to morbidity and mortality which were not investigated. Nevertheless, the employment of a large minority population bolsters the overall findings and this investigation is a solid step in providing novel and key evidence demonstrating differences in mobility performance, mobility-related SE, pain symptoms, and PA measures among OA patients with similar radiographic severity but varying weight status.

## 5. Conclusions

Exercise and weight loss are advocated in the medical management of knee OA [24] but the present findings strongly evidence the imperative nature of helping OA patients control weight. Taken in aggregate, there is clear distinction in mobility performance and PA patterns between older OA patients that differ by weight status. Older subjects who carry excessive weight are limited with regard to mobility performance and the results likely impact and are reciprocally impacted by SE outcomes, exacerbation of pain symptoms, and PA choices. Treatment options for OA patients must certainly be individualized and while self-care, patient education, therapy, exercise, and weight-loss are all considered key lines of defense against dealing with the effects of OA [25], data from IMPACT-P highlight the critical factor of weight control for all patients. As weight status increases, the burdening effect of barriers mounts. These challenges must be overcome, however, if patients are going to effectively manage their disease. Functional decline is frequently observed in longitudinal studies of knee OA patients but these challenges must be recognized and addressed in order to help OA patients through group or individual self-management of the disease.

## Figures and Tables

**Table 1:** Descriptive characteristics of the subjects (*n* = 79).

	*n*	Mean (or %)	SD	Range
Age, years	79	63.59	6.83	55–84
Weight, kilograms	79	89.57	20.74	42.27–132.27
BMI, kg/m^2^	79	32.70	7.08	18.0–48.6
Female sex	66	(83.5)		
Caucasian race	54	(68.4)		
HS graduates	77	(97.5)		
Household income less than $50,000	42	(52.5)		
Diabetes	16	(20.3)		
Hypertension	45	(57.0)		
OA elsewhere outside of the knees	79	(100)		

BMI = body mass index; HS = high school; OA = osteoarthritis.

**Table 2:** Adjusted means for mobility performance, mobility performance self-efficacy, and pain symptoms among participants of varying weight status (*n* = 79).

Variable	Normal	Overweight	Class I	Class II	Class III
Stair climb (s)	10.01* ± 1.40	10.85* ± 0.86	10.51* ± 0.91	13.10 ± 0.94	15.66 ± 1.11
400 m (s)	316.3* ± 33.5	344.7* ± 20.4	342.0* ± 21.6	376.6* ± 22.4	486.2 ± 26.5
Stair climb SE (score)	53.7 ± 9.3	46.8 ± 5.7	45.8 ± 6.0	39.7 ± 6.2	26.6 ± 7.4
400 m SE (score)	87.4* ± 8.2	76.1* ± 5.0	76.4* ± 5.3	74.5* ± 5.5	46.9 ± 6.5
WOMAC pain scale	5.82* ± 1.3	7.06* ± 0.77	7.15* ± 0.82	7.44* ± 0.85	10.38 ± 1.00

Values are the mean ± standard error; s = seconds; 400 m = 400-meter walk; SE = self-efficacy. *Denotes statistical difference from obese class III.

**Table 3:** Adjusted means for measures of physical activity among participants of varying weight status (*n* = 79).

Variable	Normal	Overweight	Class I	Class II	Class III
PA^Mod+^ (minutes)	113.4* ± 22.1	67.1^#^ ± 13.5	53.6 ± 14.3	24.3 ± 14.75	25.3 ± 17.5
Weekly steps	49,505* ± 6,138	34,229^#^ ± 3,737	35,763^∧^ ± 3,959	22,103 ± 4,096	23,175 ± 4,851
Frequency of exercise bouts >10 minutes of duration	3.16 ± 0.96	1.30 ± 0.58	1.06 ± 0.62	0.89 ± 0.64	0.79 ± 0.76

Values are the mean ± standard error; PA^Mod+^ = exercise of moderate or greater intensity. *Denotes statistical difference from obese classes I, II, and III for the outcomes. ^#^Denotes statistical difference from obese class II for the outcomes. ^∧^Denotes statistical difference from obese classes II and III for the outcome.
